# *Arabidopsis* ubiquitin ligase PUB12 interacts with and negatively regulates Chitin Elicitor Receptor Kinase 1 (CERK1)

**DOI:** 10.1371/journal.pone.0188886

**Published:** 2017-11-28

**Authors:** Koji Yamaguchi, Hirohisa Mezaki, Masayuki Fujiwara, Yuki Hara, Tsutomu Kawasaki

**Affiliations:** 1 Department of Advanced Bioscience, Graduate School of Agriculture, Kindai University, Nakamachi, Nara, Japan; 2 Institute for Advanced Biosciences, Keio University, Tsuruoka, Yamagata, Japan; National Taiwan University, TAIWAN

## Abstract

In *Arabidopsis*, fungal chitin is recognized as a pathogen-associated molecular pattern (PAMP) by the chitin receptor complex containing the lysin-motif (LysM) receptor-like kinases CERK1 and LYK5. Upon the perception of chitin, CERK1 phosphorylates the receptor-like cytoplasmic kinase, PBL27, which activates the intracellular mitogen-activated protein kinase (MAPK) cascade. However, the mechanisms by which the CERK1-PBL27 complex is regulated remain largely unknown. We identified ubiquitin ligase PUB12 as a component of the PBL27 complex using co-immunoprecipitation and mass spectrometry. However, PUB12 did not interact directly with PBL27. Instead, the ARM domains of PUB12 and its paralog PUB13 interacted with the intracellular domain of CERK1 in a manner that was dependent on its autophosphorylation, suggesting that the phosphorylation-based auto-activation of CERK1 may be required for its interaction with PUB12. The co-expression of PUB12 in *Nicotiana benthamiana* reduced the accumulation of CERK1. The *pub12 pub13* mutant exhibited enhanced chitin-induced immune responses such as ROS production, MAPK activation, and callose deposition. These results suggest that PUB12 and PUB13 are involved in the negative regulation of the chitin receptor complex, which may contribute to the transient desensitization of chitin-induced responses.

## Introduction

Plants rely on the innate immune system to defend against the invasion of potential pathogens. Immunity is initiated by the perception of pathogen-associated molecular patterns (PAMPs), including fungal chitin and bacterial flagellin. The recognition of PAMPs is mediated by plasma membrane (PM)-localized pattern recognition receptors (PRRs) [1]. PRRs are receptor-like kinases (RLKs) and receptor-like proteins (RLPs), both of which contain extracellular ectodomains potentially involved in PAMPs binding [2]. In addition to the extracellular domains, RLKs contain cytoplasmic kinase domains that appear to interact with intracellular immune components. PRR-mediated recognition triggers early immune responses such as the activation of mitogen-activated protein kinases (MAPKs) and production of reactive oxygen species (ROS), which play key steps in the induction of a number of defense responses including the synthesis of antimicrobial compounds and cell wall reinforcement.

Chitin is a major component of fungal cell walls. In *Arabidopsis*, LYK5 and CERK1, RLKs with an extracellular lysin motif (LysM), recognize chitin [3–5]. LYK5 appears to be a pseudo kinase because it lacks the critical amino acid residues for kinase activity [5]. The dimerization and autophosphorylation of CERK1 occur in response to chitin [6], and are critical steps in the activation of chitin-induced immunity [7]. Autophosphorylated CERK1 phosphorylates LYK5, leading to the endocytosis of LYK5 [8]. To activate intracellular immune responses, CERK1 phosphorylates the receptor-like cytoplasmic kinase PBL27 at PM [9]. PBL27 subsequently phosphorylates MAPKKK5 [10], which induces the rapid activation of MAPKs. Robust immune responses are mediated by the transcriptional activation of large numbers of defense-related genes through the phosphorylation of corresponding transcription factors by MAPKs [11].

Ubiquitination is one of the post-translational modification of proteins [12]. Most ubiquitinated proteins are degraded by the 26S proteasome pathway. Ubiquitin/26S proteasome-mediated protein degradation plays an important role in plant development and immunity [13]. Ubiquitination is a protein modification process that mediates the covalent binding of 76-amino acid ubiquitin peptides to target proteins, which occurs by the sequential enzymatic reactions of ubiquitin-activating enzyme (E1), ubiquitin-conjugating enzyme (E2), and ubiquitin-protein ligase (E3). The E3 ligases have been classified into three classes: HECT (homologous to the E6-associated protein C terminus), RING (a really interesting new gene) finger, and U-box [12]. Recent studies on plant ubiquitin E3 ligases indicated that plants contain a large number of U-box-type ubiquitin ligases (PUB; plant U-box) that positively or negatively regulate innate immunity in plants [14].

Excessive or prolonged defense responses are detrimental to plant cells and cause plant retardation. Therefore, the down-regulation of immune signaling is crucial for the proper control of immunity. Recent findings suggest that the attenuation of immunity is regulated by the inhibition of PRR activation or degradation of PRRs [15] [16]. *Arabidopsis* PRR FLS2 recognizes flagellin-derived 22-amino acid peptide (flg22) with co-receptor BAK1 [2], which induces the intracellular activation of immune responses. *Arabidopsis* protein phosphatase type 2A (PP2A) constitutively interacts with and negatively regulates BAK1 activity in the absence of flg22. PP2A activity is reduced upon the perception of flg22, which results in the activation of BAK1 [16]. BAK1 phosphorylates the *Arabidopsis* U-box ubiquitin E3 ligases PUB12 and PUB13 [17], which stimulate the association between FLS2 and PUB12/PUB13. Consequently, FLS2 is ubiquitinated by PUB12/PUB13 and then degraded through a 26S proteasome pathway. In a similar manner, rice PRR Xa21 is negatively regulated by the protein phosphatase 2C and ubiquitinated by the RING-finger ubiquitin E3 ligase XB3 [18]. However, in contrast to PUB12/PUB13, XB3 positively regulates Xa21-mediated immunity.

As described above, CERK1 and LYK5 form a receptor complex for the perception of chitin. Liao et al. recently reported that CERK1 was degraded by a treatment with chitin [19]. In contrast to this finding, Erwig et al. showed that CERK1 was strongly phosphorylated in response to chitin; however, the protein level of CERK1 was not altered at PM [8]. CERK1 is known to constitutively undergo ectodomain shedding [20]. In animal tyrosine kinase receptors, ectodomain shedding generates soluble intracellular kinase domains that are often degraded by the proteasome-dependent pathway [21]. Thus, the protein level of CERK1 may be constitutively regulated by ectodomain shedding and the proteasome-dependent protein degradation of the kinase domain. In contrast to CERK1, the perception of chitin triggers LYK5 endocytosis [8], which depends on the CERK1-mediated phosphorylation of LYK5. Since LYK5 is known to be ubiquitinated by PUB13 [19], ubiquitination may induce the internalization of LYK5 and its degradation in vacuoles.

In the present study, we identified PUB12 as a component of the PBL27-containing complex. However, PUB12 did not directly interact with PBL27. A yeast two-hybrid assay showed that PUB12 interacted with the intracellular domain of CERK1, not LYK5. PUB13 also showed a weak interaction with the intracellular domain of CERK1. The interaction between PUB12 and the intracellular domain of CERK1 depended upon the kinase activity of CERK1. The co-expression of PUB12 and CERK1 reduced the protein level of CERK1 in *Nicotiana benthamiana*. Chitin-induced immune responses were up-regulated in the *pub12/pub13* mutant. These results indicate that PUB12 negatively regulates chitin-induced immune responses, possibly through the interaction with CERK1.

## Materials & methods

### Plant materials

*Arabidopsis* plants were grown in a 16 h light/ 8 h dark cycle at 22°C (light) or 20°C (dark). PBL27-3HA plant was described previously [9].

### Plasmid

DNA fragments of *PUB12* and *PUB13* were PCR-amplified using gene-specific primers (S2 Table), and ligated into a pENTR/D-TOPO vector (Invitrogen). pENTR/D-TOPO-CERK1^D441V^ and PBL27 were described previously (Shinya et al., 2014). For the transient expression in *Nb* leaves, the *PUB12* coding regions in pENTR/D-TOPO were transferred into pGWB6. The *CERK1* and *CERK1*^*D441V*^ coding regions in pENTR/D-TOPO were transferred into pGWB14.

### Mass spectrometry

The PBL27 protein complex immunoprecipitated with α-HA was separated using ready-made 12.5% (w/v) SDS-polyacrylamide gels and subjected to liquid chromatography-tandem mass spectrometry (LTQ-Orbitrap XL: Thermo Fisher Scientific). Trypsin digestion and a mass spectrometric analysis were conducted as described previously [22]. The spectra obtained were compared with a protein database (TAIR10) using the MASCOT server (version 2.4).

### Yeast two hybrid assay

Yeast two-hybrid assays were performed according to the yeast protocol handbook (Clontech, http://www.clontech.com/). DNA fragments of *PBL27*, *PUB12*, *PUB13*, *CERK1*-intracellular domain (IC), *CERK1* ^*D441V*^ -IC, and *LYK5*-IC were transferred into the vectors pBTM116 (bait vector) and pVP16 (prey vector). The yeast two-hybrid interaction was analyzed based on the requirement for histidine for yeast growth as described previously (Ishikawa et al., 2014). To analyze protein levels in yeast, yeast cells were resuspended in 100 μl PBS buffer (140 mM NaCl, 2.7 mM KCl, 6.5 mM Na_2_HPO_4_, and 1.5 mM KH_2_PO_4_ pH7.4). After the addition of 20 μl of SDS sample buffer (350 mM Tris-HCl (pH 6.8), 10% SDS, 3% glycerol, 600 mM DTT, and 0.012% bromophenol blue), protein extracts were incubated at 95°C for 10 min. Proteins were detected by immunoblots with α-VP16 or α-LexA (Santa Cruz Biotechnology) using ImageQuant LAS4000 (GE Healthcare). To detect CERK1 phosphorylation in yeast cells, proteins were separated by SDS-PAGE on 7.5% acrylamide gels containing 20 μM Phos-tag Acrylamide (Wako Pure Chemical Industries). The following procedure was performed according to the manufacturer’s protocol.

### Transient expression in *Nb* leaves

Constructs for transient expression were transformed into *A*. *tumefaciens* strain GV3101 harboring the helper plasmid pSoup. Transformed *A*. *tumefaciens* in buffer containing 10 mM MgCl_2_, 10 mM 2-(N-morpholine)-ethanesulfonic acid (MES)-NaOH, pH 5.6, and 150 μM acetosyringone was syringe-infiltrated into *Nb* leaves as reported previously [9]. The silencing suppressor p19 (OD600 = 0.05) was co-expressed in all experiments.

### Ion leakage analysis

Twenty-three hours after the inoculation, leaf discs (8.5 mm in dimeter) were transferred to a Petri dish containing 30 mL of purified water for 1 h. Ten leaf discs were then transferred to plastic tubes containing 12 mL of purified water. The conductivity of the samples was assessed using a portable conductivity meter (Yokogawa Electric Corporation).

### Protein analysis

*Nb* leaves were ground in liquid nitrogen and resuspended in extraction buffer (50 mM Tris-HCl (pH 7.5), 150 mM NaCl, 10% glycerol, 5 mM DTT, 2.5 mM NaF, 1.5 mM Na_3_VO_4_, 1x Complete EDTA free protease inhibitor cocktail (Roche), and 2% (v/v) IGEPAL CA-630 (MP Biomedicals)). In the immunoprecipitation assay, the supernatant was incubated with anti-HA magnetic beads (Miltenyi Biotec). The beads were washed four times with the extraction buffer and resuspended in an equal volume of 2× SDS sample buffer. Co-immunoprecipitated proteins were analyzed by immunoblots. To purify the microsomal fraction, tissue was homogenized on ice with extraction buffer 1 (20 mM Tris-HCl pH7.5, 0.33 M sucrose, 1 mM EDTA, protease inhibitor cocktail (Roche), and 0.1% 2-Me-OH). Crude protein extracts were centrifuged at 2000 x *g* to remove cell debris, and the supernatants were then centrifuged at 82,300 x *g* for 1 h to pellet microsomal fractions. Supernatants were used as soluble proteins. Pellets were suspended in extraction buffer 2 (50 mM Tris-HCl (pH 7.5), 150 mM NaCl, 10% glycerol, 5 mM DTT, 2.5 mM NaF, 1.5 mM Na_3_VO_4_, 1x Complete EDTA free protease inhibitor cocktail (Roche), and 1% (v/v) IGEPAL CA-630 (MP Biomedicals)) and used as microsomal proteins. α-PIP1 (Agrisera) was used as a PM-localized protein marker.

### Chitin-induced immune assay

*Arabidopsis* seeds were surface-sterilized and germinated in MGRL medium (Naito et al., 1994) containing 0.1% agarose and 1% sucrose. Seedlings were grown for 8–10 days in a 16 h light/ 8 h dark cycle at 22°C (light) or 20°C (dark). The seedlings were pre-incubated in liquid MGRL medium for 3 hours and then treated with 10 μM (GlcNAc)_7_. Experiments for MAPK activation, ROS production, and callose deposition were performed as described previously [10].

### *In vitro* ubiquitination assay

The CERK1-HA, AtUBC8 and PUB12 were subcloned into a pCold GST vector (Takara). The recombinant proteins were expressed using a Cold-Shock bacterial expression system (Takara), and purified using Glutathione Sepharose 4B (GE Healthcare). The CERK1-HA and AtUBC8 proteins were purified by on columm Turbo3C Protease (Accelagen) digestion. The *in vitro* ubiquitination assays were performed as described with some modifications [17]. The reactions contained 1μg of purified CERK1-HA, 500 ng of His6-E1(human UBE1) (Boston Biochem), 1μg of purified E2 (AtUBC8), 2.5 μg of FLAG tagged ubiquitin (Boston Biochem) and 4μg of purified GST-PUB12 in the ubiquitination buffer (0.1 M Tris-HCl pH 7.5, 25 mM MgCl2, 2.5 mM dithiothreitol, 10 mM ATP) to a final volume of 60 μl. The reactions were incubated at 30°C for 2 hr, and then stopped by adding SDS sample buffer and boiled at 100°C for 5 min. The samples were then separated by SDS–PAGE and the ubiquitinated substrates were detected by Western blotting analysis using an anti-FLAG antibody (F3165; Sigma-Aldrich) or an anti-HA antibody (11867423001; Roche).

## Results

### Identification of components of the PBL27 complex

**Fig 1:**
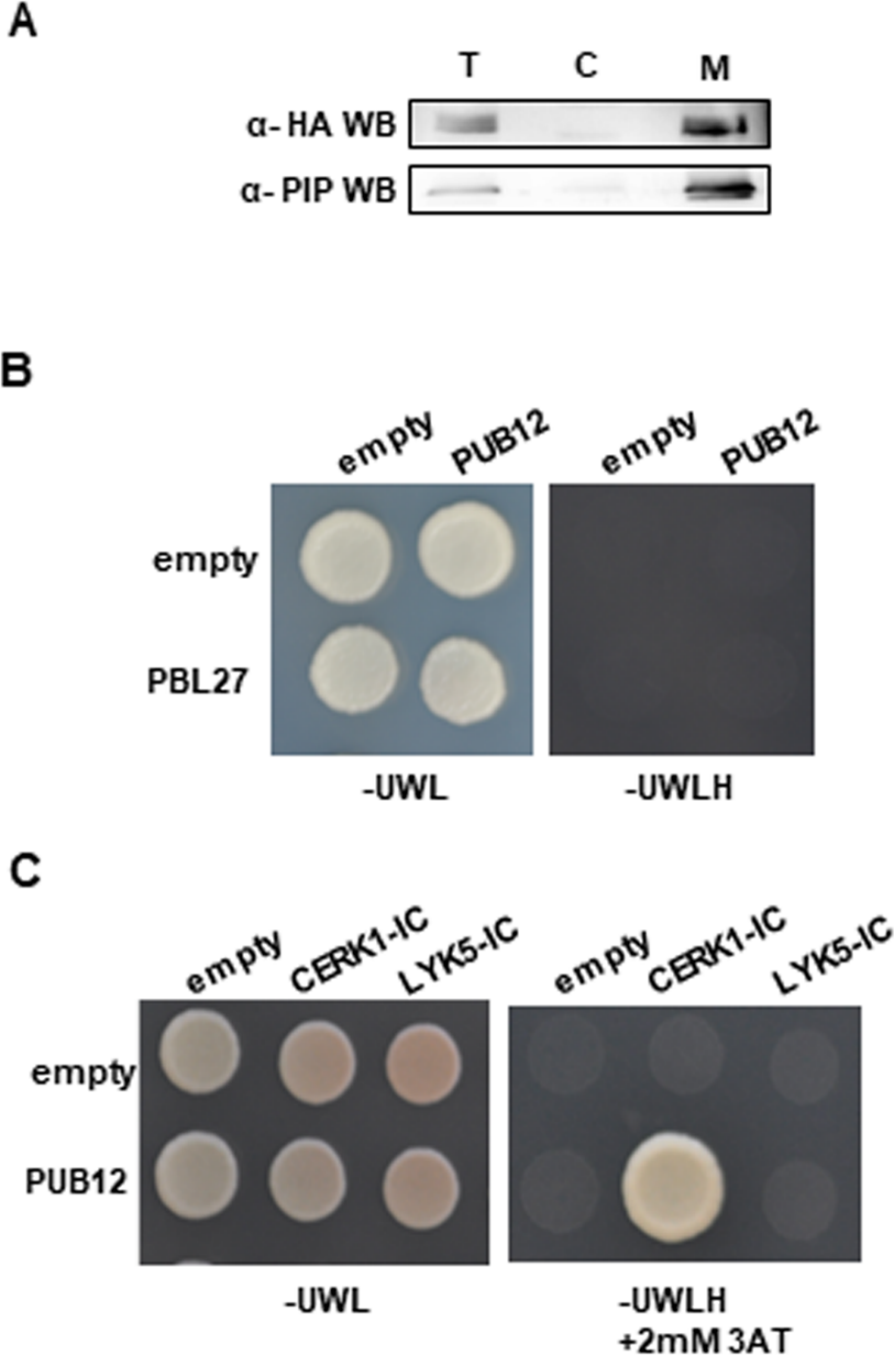
Identification of PUB12 in the PBL27 complex. (A) The PBL27-3HA protein was detected in the membrane fraction. The total (T), soluble (S), and microsomal membrane (M) fractions purified from *Arabidopsis* seedlings expressing PBL27-3HA were used in immunoblots with α-HA. (B) PBL27 does not interact with PUB12 in the yeast two hybrid experiment. The growth of yeast colonies on the plate. (C) PUB12 interacts with the intracellular domain (IC) of CERK1, but not LYK5 in the yeast two hybrid experiment. The growth of yeast colonies on plates (-ULWH) lacking uracil (U), leucine (L), tryptophan (W), and histidine (H) with 2 mM 3-aminotriazole (3-AT) indicates a positive interaction.

To identify immune components included in the PBL27 complex, we generated transgenic plants expressing C-terminal 3xHA-tagged PBL27 (PBL27-HA) under the control of native promoter. We previously reported that PBL27 was localized at PM when transiently expressed in *N*. *benthamiana* (*Nb*) [9]. To confirm the subcellular localization of PBL27 in *Arabidopsis*, soluble fractions and microsomal fractions were prepared from PBL27-HA plants, and subjected to immunoblots with α-HA (Fig 1A). PBL27-HA was localized in the membrane fraction, but not the soluble fraction, which was consistent with previous findings of its PM localization in *Nb* leaves [9].

To identify the components included in the PBL27 complex, protein extracts prepared from PBL27-3HA were immunoprecipitated with α-HA, and subjected to liquid chromatography-tandem mass spectrometry. We identified 4 proteins as candidates for components included in the PBL27 complex (S1 Table). Of these, we selected the U-box-type ubiquitin ligase PUB12 for further analyses because it was previously reported to be located in the FLS2/BAK1 immune receptor complex at PM [17].

### PUB12 interacts with CERK1

PUB12 encodes the E3 ubiquitin ligase with a U-box domain and C-terminal armadillo (ARM) repeat domain [17]. To examine whether PUB12 directly interacts with PBL27, we performed a yeast two hybrid experiment (Fig 1B). However, an interaction was not observed between PUB12 and PBL27. Since PUB12 directly interacts with the intracellular kinase domain of BAK1 [17], we analyzed the interaction of PUB12 with the kinase domains of CERK1 and LYK5. The yeast two-hybrid assay showed that PUB12 interacted with the intracellular domain of CERK1, but not LYK5 (Fig 1C).

**Fig 2:**
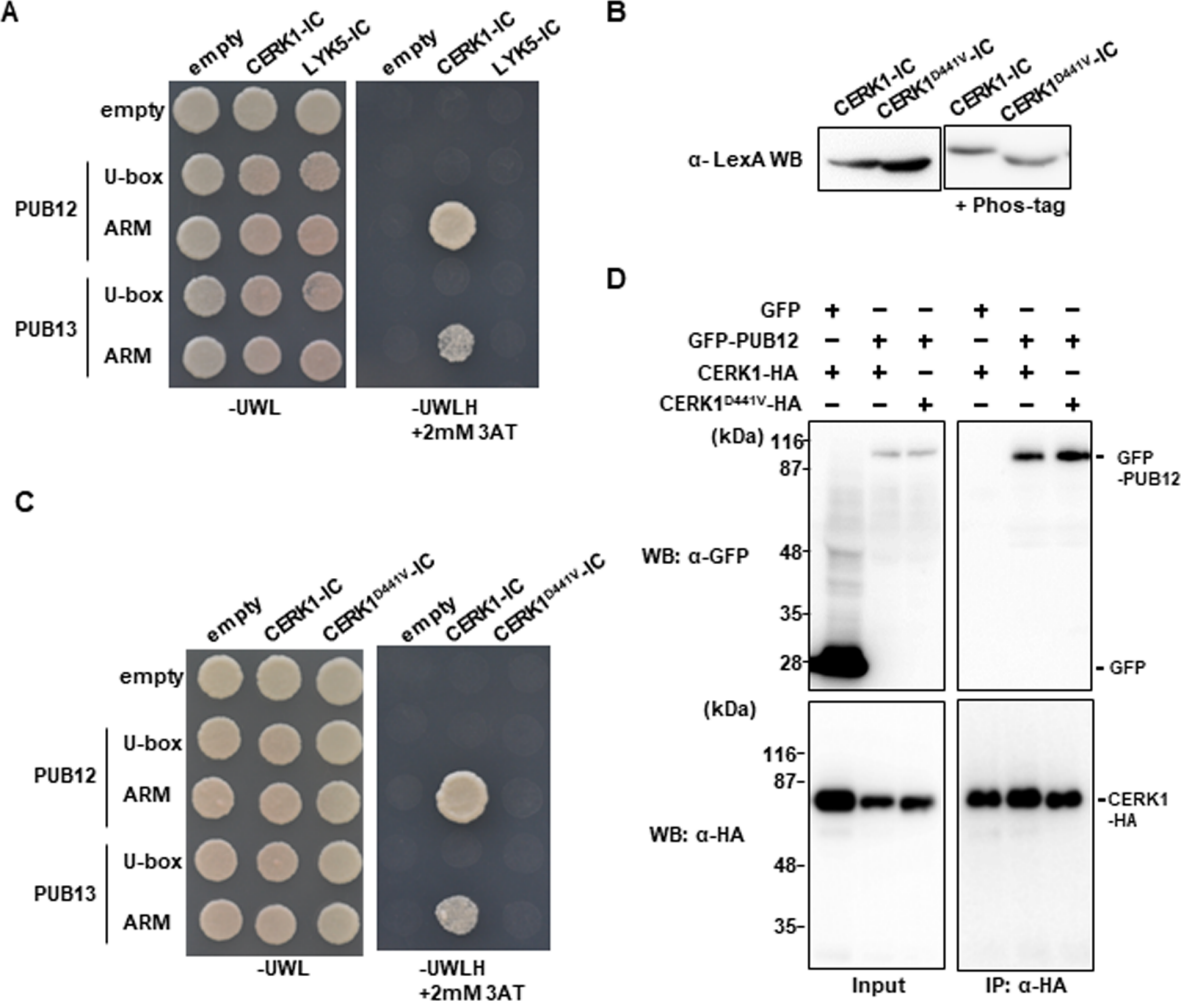
Interaction of PUB12 and PUB13 with CERK1 depends on its kinase activity. (A) PUB12 and PUB13 interact with the intracellular domain (IC) of CERK1, but not LYK5 in the yeast two hybrid experiment. The growth of yeast colonies on plates (-ULWH) lacking uracil (U), leucine (L), tryptophan (W), and histidine (H) with 2 mM 3-aminotriazole (3-AT) indicates a positive interaction. (B) Immunoblot analysis of yeast extracts containing LexA fusion proteins. Total proteins were prepared from the yeast cells used for the two-hybrid assays in Fig 1B. Proteins were subjected to Phos-tag SDS-PAGE (right) or SDS-PAGE (left). Gels were analyzed by Western blotting with α-LexA. (C) PUB12 and PUB13 interact with CERK1-IC, but not the kinase-inactive mutant (CERK1^D441V^) in the yeast two hybrid experiment. (D) Co-immunoprecipitation assays show that PUB12-GFP forms a complex with the kinase-active and inactive forms of CERK1 in *Nb* leaves.

To identify which domains of PUB12 are responsible for the interaction with CERK1, we divided two regions, including the U-box domain or ARM domain, and performed yeast two-hybrid experiments (Fig 2A). The ARM domain, but not U-box domain specifically interacted with the intracellular domain of CERK1. We also examined the interaction between PUB13 and the intracellular domain of CERK1. PUB13 is highly homologous to PUB12 (89% similarity at the amino acid level). Although the ARM domain of PUB13 bound to the intracellular domain of CERK1 (Fig 2A), the interaction between PUB13 and the intracellular domain of CERK1 was markedly weaker than that with PUB12.

In response to the perception of chitin, CERK1 becomes phosphorylated at multiple residues in the juxtamembrane and kinase domains [7]. If PUB12 and PUB13 recognize the activation of CERK1, they may be able to distinguish between the inactive (unphosphorylated) and active (phosphorylated) states of CERK1. We generated a kinase-inactive CERK1^D441V^ mutant by the substitution of aspartic acid at position 441, located at the active site of the kinase domain, to valine, and analyzed the interaction with PUB12 and PUB13. The intracellular domain of CERK1 ^D441V^ was not phosphorylated in yeast, whereas the intracellular domain of wild-type CERK1 was, possibly by its auto-phosphorylation activity (Fig 2B). The ARM domains of PUB12 and PUB13 did not interact with the intracellular domain of CERK1 ^D441V^ (Fig 2C), indicating that PUB12 and PUB13 recognize the phosphorylated state of CERK1.

To analyze the *in vivo* interaction between CERK1 and PUB12, N-terminal GFP-fused PUB12 (GFP-PUB12) and C-terminal 3xHA-tagged CERK1 (CERK1-HA) were transiently expressed in *Nb* leaves, and subjected to co-immunoprecipitation assays. GFP-PUB12 was co-immunoprecipitated with the wild-type and kinase-inactive forms of CERK1 (Fig 2D). These results suggested that PUB12 constantly formed a complex with CERK1.

### Co-expression of PUB12 reduces CERK1 abundance in *Nb* leaves

**Fig 3:**
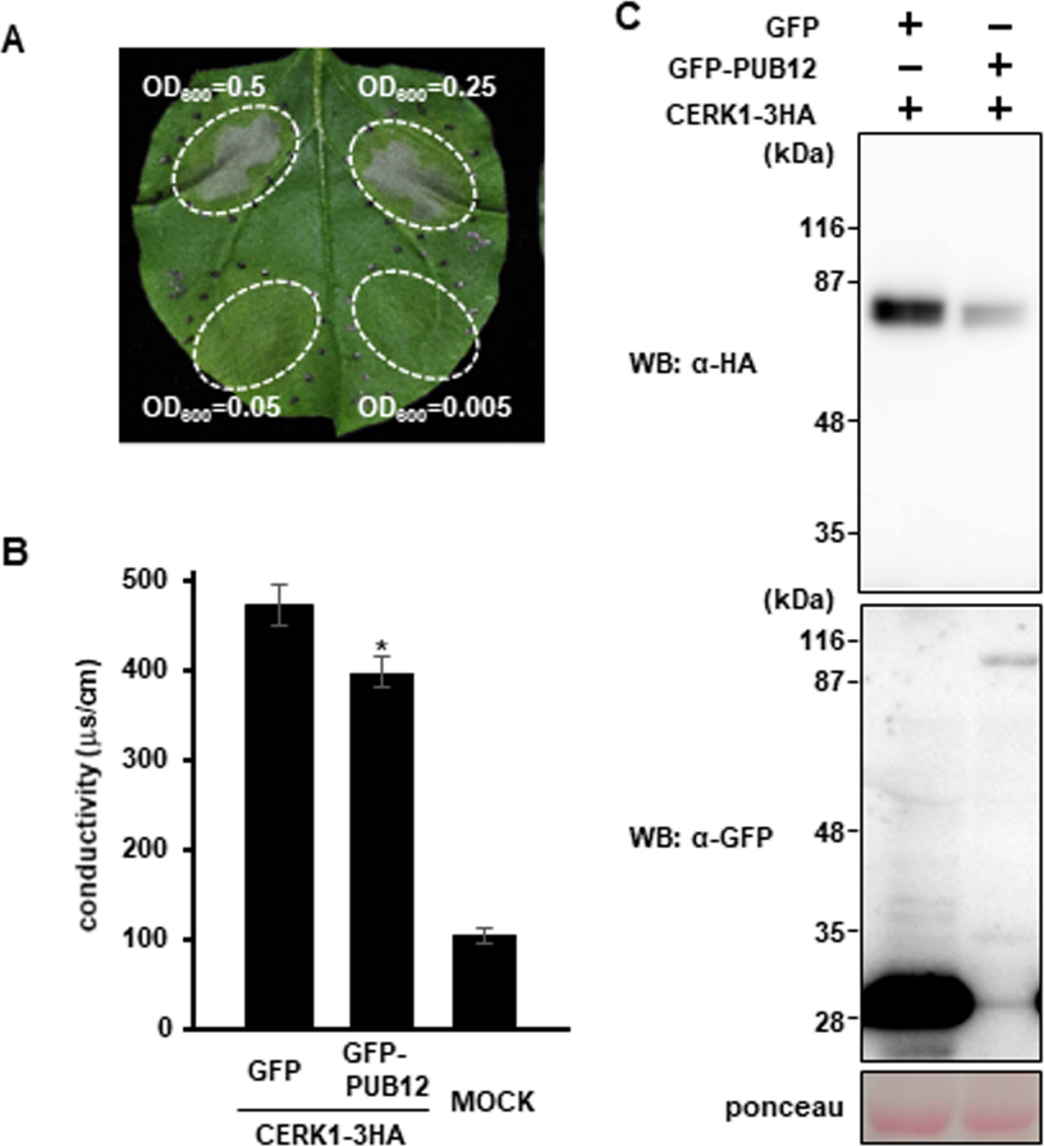
Expression of PUB12 reduces CERK1 protein levels. (A) The overexpression of CERK1 induces pathogen-independent cell death in *Nb* leaves. Cell death in infiltrated *Nb* leaf areas overexpressing CERK1-10MYC was assessed 3 days post-infiltration (dpi). (B) PUB12 suppresses CERK1-induced cell death. Ion leakage in the infiltrated leaf areas co-expressing GFP or GFP-PUB12 with CERK1-3HA was measured at 2 dpi. Infiltration was performed using a blunt syringe at OD600 = 0.5 for all samples. The MOCK treatment with water was used as an internal control. Data are the mean ± SE (n = 10). The asterisk indicates significant differences from GFP by the Student’s *t*-test (*P* < 0.05). (C) Reductions in CERK1 protein levels by PUB12. *Nb* leaves were infiltrated with *Agrobacterium tumefaciens* strains to co-express GFP (OD600 = 0.05) or GFP-PUB12 (OD600 = 0.5) with CERK1-3HA (OD600 = 0.05). Protein was extracted from leaf samples 36 hours post-infiltration.

To elucidate the biological relevance of the interaction between PUB12 and CERK1, we transiently co-expressed CERK1-HA with GFP-PUB12 in *Nb* leaves. The transient expression of CERK1 induced cell death in *Nb* leaves (Fig 3A), which was consistent with previous findings [23]. The co-expression of GFP-PUB12 with CERK1-HA also induced cell death. Since the levels of cell death induced with or without GFP-PUB12 were not distinguished by visible observations, we quantified cell death levels by measuring electrolyte leakage. The co-expression of GFP-PUB12 with CERK1-HA resulted in significantly less ion leakage than CERK1-HA alone (Fig 3B). Therefore, we investigated whether GFP-PUB12 affects the protein level of CERK1-HA. Immunoblotting experiments indicated that the expression of GFP-PUB12 reduced the protein abundance of CERK1-HA (Fig 3C). Thus, the reduction observed in CERK1-induced cell death appeared to be caused by the lower abundance of CERK1.

### PUB12 and PUB13 redundantly regulate chitin-induced immune responses

**Fig 4:**
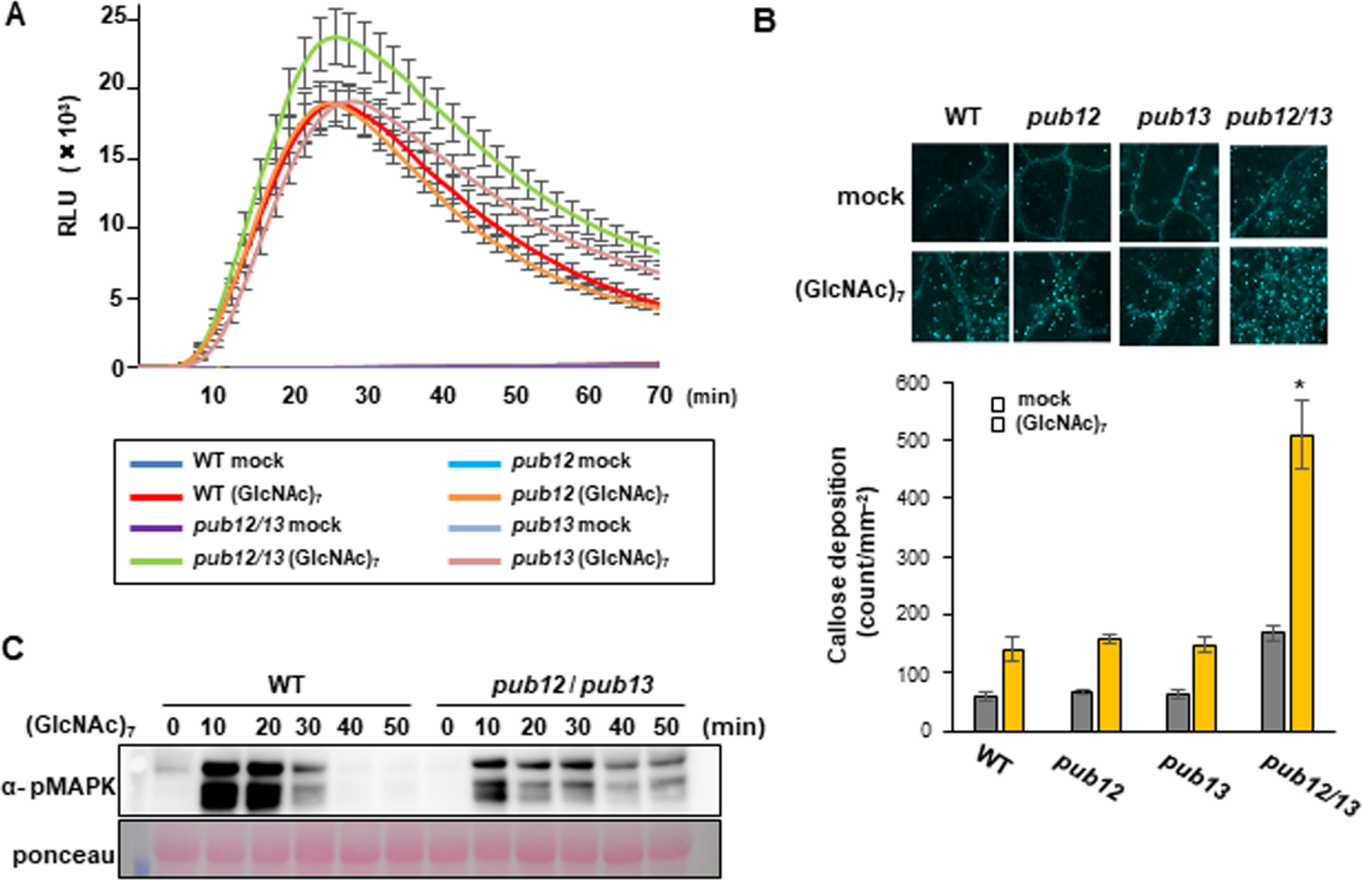
*pub12* and *pub13* double mutations enhance chitin-induced immunity. (A) ROS production by 10-day-old seedlings following a treatment with 10 μM (GlcNAc) _7_. Data are the mean ± SE (n = 10). (B) Chitin-induced callose deposition in the *pub12*, *pub13*, and *pub12/13* mutants. Ten-day-old seedlings were treated with 10 μM (GlcNAc) _7_ for 18 h. Data represent the mean of 11 cotyledons ± SE. The asterisks indicate significant differences from the wild-type (WT) controls by Welch’s *t*-test (*P* < 0.01). (C) Chitin induced MAPK activation in *pub12/pub13* mutants. Total proteins were extracted from seedlings treated with 10 μM (GlcNAc) _7_. The activation of each MAPK was analyzed by immunoblots with α-pMAPK. The protein loading control was shown by Ponceau staining. Experiments were repeated at least three times with similar results.

Since PUB12 and/or PUB13 interact with CERK1, chitin ((GlcNAc)_7_)-induced immunity may be influenced by *pub12* and/or *pub13* mutations. Therefore, we analyzed chitin-induced immune responses. Chitin-induced ROS production was measured using chemiluminescence mediated by L-012. The ROS levels of the *pub12* and *pub13* single mutants were similar to those of the wild type (Fig 4A). However, ROS production was significantly enhanced in the *pub12*/*pub13* double mutant. Similarly, chitin-induced callose deposition increased in the *pub12*/*pub13* mutant, but not in the single mutants (Fig 4B). We analyzed the chitin-induced activation of the MAP kinases MPK3 and MPK6 using an anti-pMAPK antibody. The *pub12*/*pub13* double mutant exhibited prolonged activation (Fig 4C). Thus, PUB12 and PUB13 appear to redundantly and negatively regulate ROS production, callose deposition, and MAPK activation in chitin signaling.

## Discussion

The negative regulation of immune responses is a crucial step in preventing growth retardation [2]. One mechanism by which this is achieved is the down-regulation of PRRs after signal activation [24]. Recent studies indicated that PRRs form a homodimer or heterodimer in response to ligands and transphosphorylation occurs between the cytoplasmic kinase domains of PRRs [2], suggesting that transphosphorylation is a key step in PRR activation. Kinase-inactive mutants of PRRs cannot activate immune responses, even in the presence of their ligands [25]. We herein demonstrated that the ARM domains of PUB12 and PUB13 interacted with kinase-active CERK1, but not kinase-inactive CERK1. This result suggests that PUB12 and PUB13 recognize activated (phosphorylated) CERK1 based on its phosphorylation status. In addition, although PUB12 did not directly bind to kinase-inactive CERK1^D441V^ in the yeast two-hybrid system, CERK1 ^D441V^ was co-immunoprecipitated with PUB12. These results imply that PUB12 and unphosphorylated CERK1 are located close to each other in the complex, but do not directly interact. Since the perception of chitin induces the phosphorylation of CERK1, this phosphorylation may trigger a direct interaction between CERK1 and PUB12.

Since the ARM domain of PUB12 interacted with the intracellular domain of CERK1, we tested whether PUB12 ubiquitinates CERK1 using an *in vitro* ubiquitination assay. However, we failed to detect the ubiquitination of CERK1 by PUB12 even though the strong self-ubiquitination of PUB12 was observed. Therefore, it is possible that the protein level of CERK1 may be regulate indirectly by PUB12. Other components may also be essential for the PUB12-mediated ubiquitination of CERK1.

CERK1 has been shown to constitutively undergo ectodomain shedding [20]. Ectodomain shedding has been suggested to generate a soluble intracellular kinase domain [21]. Interestingly, the ectodomain of CERK1 accumulates in response to fungal infection [20]. Since the ARM domain of PUB12 interacts with the intracellular domain of CERK1, the intracellular domain generated by ectodomain shedding increased during pathogen infection may be degraded by PUB12-mediated ubiquitination. It currently remains unclear whether *pub12*/*pub13* mutations result in the accumulation of a soluble kinase domain because an antibody for the detection of the kinase domain is not yet available.

The *pub12*/*pub13* double mutation enhanced chitin-induced ROS production and callose deposition. However, single *pub12* or *pub13* mutations did not affect these immune responses, suggesting that PUB12 and PUB13 redundantly function in chitin signaling, as found in flg22-induced immunity [17]. In contrast to our result, Liao et al. reported that chitin-induced ROS production was enhanced in a *pub13* single mutant [19], and this discrepancy may have been due to a difference in the experimental systems used, such as chitin treatments and plant ages. These enhanced chitin responses in *pub12*/*pub13* indicate that PUB12 and PUB13 negatively regulate the chitin receptor complex. Further investigations on the spatio-temporal dynamics of CERK1 in *pub12* and/or *pub13* and the identification of other components of the CERK1/LYK5 complex are needed to obtain a clearer understanding of the roles of PUB12 and PUB13 in chitin signaling.

## Supporting information

S1 Table Identification of the components of PBL27complex by mass spectrometry. (TIF)

S2 Table Primers used in study. (TIF)
