# Supplementary figures and images for: Arabidopsis ubiquitin ligase PUB12 interacts with and negatively regulates Chitin Elicitor Receptor Kinase 1 (CERK1)
Source: PLoS One. 2017 Nov 28;12(11):e0188886. doi: 10.1371/journal.pone.0188886 (PMC5705137; doi:10.1371/journal.pone.0188886)

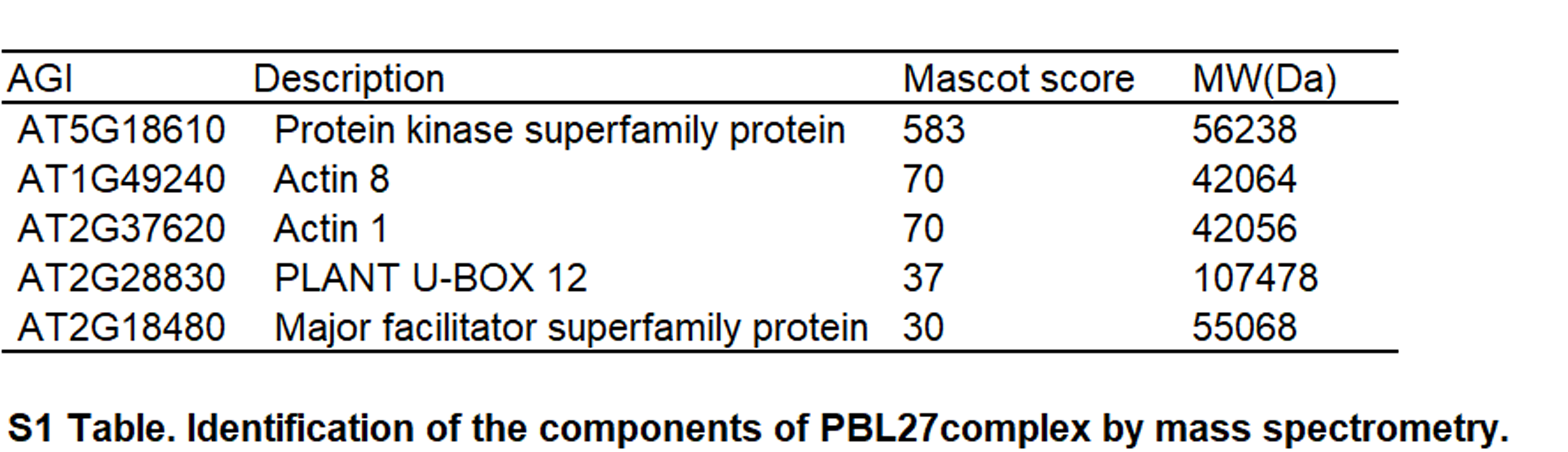

Supplement: S1 Table — (TIF) [file pone.0188886.s001.tif]

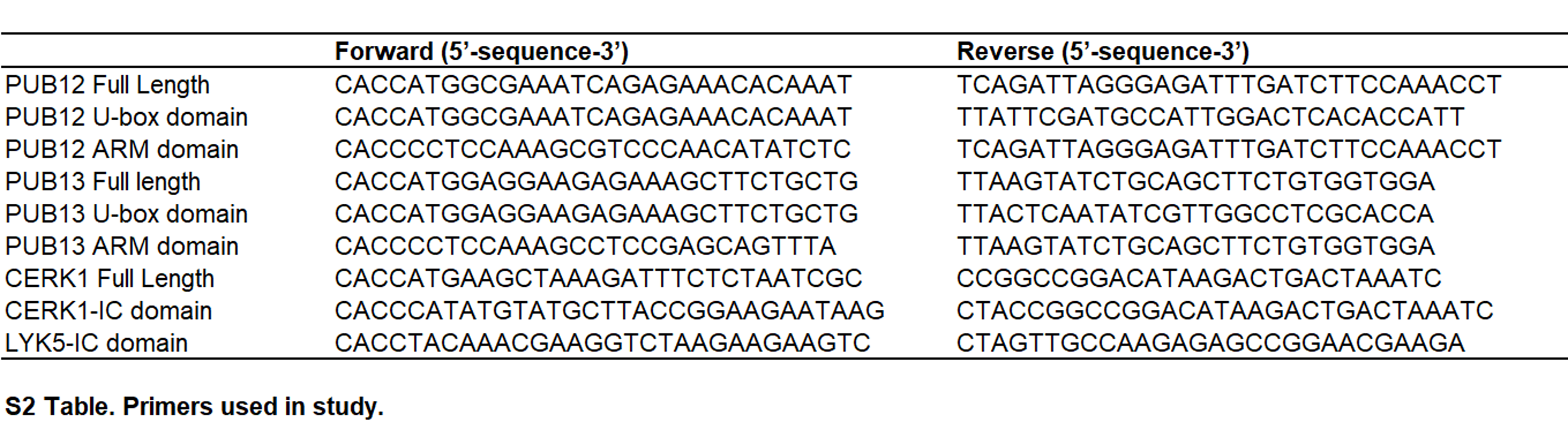

Supplement: S2 Table — (TIF) [file pone.0188886.s002.tif]
